# Correction: N-Terminal Polypeptide of Annexin A2 Decreases Infection of *Mycoplasma hyorhinis* to Gastric Cancer Cells

**DOI:** 10.1371/journal.pone.0153995

**Published:** 2016-04-14

**Authors:** The PLOS ONE Staff

## Notice of Republication

This article was republished on April 6, 2016, as all instances of the character μ were erroneously dropped during the typesetting of the article for publication. The publisher apologizes for the errors.

Please download this article again to view the correct version. The originally published, uncorrected article and the republished, corrected articles are provided here for reference.

## Supporting Information

S1 File Originally published, uncorrected article. (PDF)

S2 File Republished, corrected article. (PDF)

## References

[1] Yuan S, Qu L, Shou C (2016) N-Terminal Polypeptide of Annexin A2 Decreases Infection of *Mycoplasma hyorhinis* to Gastric Cancer Cells. PLoS ONE 11(1): e0147776 doi: 10.1371/journal.pone.0147776 26812398 10.1371/journal.pone.0147776 PMC4727897

