# Supplementary material for: Correction: N-Terminal Polypeptide of Annexin A2 Decreases Infection of Mycoplasma hyorhinis to Gastric Cancer Cells
Source: PLoS One. 2016 Apr 14;11(4):e0153995. doi: 10.1371/journal.pone.0153995 (PMC4831741; doi:10.1371/journal.pone.0153995)
Supplement: S2 File — (PDF) [file pone.0153995.s002.PDF]

PLOS ONE

RESEARCH ARTICLE

N-Terminal Polypeptide of Annexin A2 Decreases Infection of *Mycoplasma hyorhinis* to Gastric Cancer Cells

Shiqin Yuan, Like Qu, Chengchao Shou*

Key Laboratory of Carcinogenesis and Translational Research (Ministry of Education), Department of Biochemistry and Molecular Biology, Peking University Cancer Hospital & Institute, Beijing, China

* cshou@vip.sina.com

CrossMark
click for updates

OPEN ACCESS

Citation: Yuan S, Qu L, Shou C (2016) N-Terminal Polypeptide of Annexin A2 Decreases Infection of *Mycoplasma hyorhinis* to Gastric Cancer Cells. PLoS ONE 11(1): e0147776. doi:10.1371/journal.pone.0147776

Editor: Gernot Zissel, Universitatsklinikum Freiburg, GERMANY

Received: October 16, 2015

Accepted: January 7, 2016

Published: January 26, 2016

Copyright: © 2016 Yuan et al. This is an open access article distributed under the terms of the Creative Commons Attribution License, which permits unrestricted use, distribution, and reproduction in any medium, provided the original author and source are credited.

Data Availability Statement: All relevant data are within the paper. Microarray data has been deposited in NCBI Gene Expression Omnibus (GEO) (accession no.GSE73777).

Funding: Funded by National Natural Science Foundation of China (81572532). http://www.nsfc.gov.cn/publish/portal1/. SCC received the funding. The funders had no role in study design, data collection and analysis, decision to publish, or preparation of manuscript.

Competing Interests: The authors have declared that no competing interests exist.

Abstract

Mycoplasma infection in human and its contamination in cell cultures are worldwide problems. The drugs currently available for preventing or treating mycoplasma infection suffer from low sensitivity, strong resistance and high toxicity. Our previous work showed that *Mycoplasma hyorhinis* (*M*. *hyorhinis*) infection was mediated by the interaction between p37 of *M*. *hyorhinis* and Annexin A2 (ANXA2) of host cells, however the translational value of this mechanism was unknown. Herein, we synthesized the N-terminal of ANXA2 polypeptide (A2PP) and found that A2PP could decrease the infection of *M*. *hyorhinis* to gastric cancer cells and block *M*. *hyorhinis* infection-induced cell migration. Furthermore, we found that A2PP could reduce *M*. *hyorhinis* contamination of passage cells. Moreover, compared with the commercial antibiotics commonly used in cell culture to prevent *M*. *hyorhinis* infection, A2PP demonstrated a more effectiveness but a low toxicity on cell growth. Thus, our study for the first time revealed A2PP's potential for the treatment and prevention of *M*. *hyorhinis* infection.

Introduction

Pathogenic mycoplasmas, including Mycoplasma pneumoniae (*M*. *pneumoniae*), mycoplasma hyorhinis (*M*. *hyorhinis*), oral mycoplasma (*M*.*orale*) and Mycoplasma genitalium (*M*. *genitalium*), belong to class mollicutes, which is the smallest microorganism living in nature and can duplicate independently [1–2]. Many studies showed that infection with these mycoplasmas was associated with tumor development [3–8]. *M*. *orale* causes chromosome abnormality in human diploid cells and induces cell transformation [6]. Infection with *M*. *hyorhinis* or *M*. *genitalium* could increase the migration and invasiveness of prostate epithelial cells [3]. *M*. *hyorhinis* infection has been linked to arthritis, serositis, infertility and cancer of human [9–12].

Additionally, mycoplasma contamination in cell culture is a serious problem and the rate of passage cells infected by mycoplasma is high. Cell culture is widely used in life sciences, such as in the basic research, clinical trial research, development and production of biological products, as well as in the field of biopharmaceutical and vaccine production. Preventing the cells from

microbial contamination is critical to ensure the quality of research. The mycoplasma contamination is the most common problem in cell culture with an incidence of 30%-60% [1]. It was shown that four species of mycoplasmas account for more than 95% of infection in cell culture, including *M.orale*, arginine mycoplasma (*M. arginini*), *M. hyorhinis*, and Levin's no Acholeplasma (*A. laidlawii*) [13,14,15,16]. Because of small size, mycoplasma is hardly to be found under the light microscope and is easily ignored by researchers. In recent years, numerous antibiotics used for mycoplasma treatment repeatedly resulted in the emergence of mycoplasma resistance. For these reasons, it is imperative to develop new agents for preventing or decreasing mycoplasma infection.

Our previous work showed that *M. hyorhinis* infection depends on the interaction of p37 (major membrane protein of *M. hyorhinis*) and host ANXA2 through their N-terminal domains. We also found polyclonal antibody to p37 could block the infection of *M. hyorhinis* and decrease *M. hyorhinis*-promoted migration of gastric cancer cell [17]. Based on these discoveries, we raised a hypothesis that the peptide, synthesized according to the amino acid sequence of ANXA2 N-terminal region (from 1st to 30th aa, here we named this peptide as A2PP), could block *M. hyorhinis* infection via its competition with ANXA2 and possibly be used as a drug for preventing *M. hyorhinis* infection in cell culture. In this study, we tested this hypothesis and also compared the effects of A2PP with other drugs in preventing *M. hyorhinis* infection.

Materials and Methods

Cell Culture

AGS gastric cancer cell line was from American Type Culture Collection (ATCC). BGC823 gastric cancer cell line was established by the Peking University People's Hospital and was purchased from Cell Culture Center of Chinese Academy of Medical Sciences (Beijing, China). AGS and BGC823 cells were cultured in RPMI-1640 medium supplemented 10% fetal calf serum obtained from Invitrogen (Carlssbab, CA, USA). Mycoplasma test was implemented before each new experiment by PCR amplification of *M. hyorhinis p37*.

Mycoplasma Propagation and Co-Culture

Mycoplasma propagation and co-culture was carried out as previously reported [17,18,19].

Antibodies and Reagents

Anti-p37 monoclonal antibody PD4 was generated and characterized previously [5,20,21]. Polyclonal anti-p37 antibody was obtained from immunizing rabbit with GST-p37 fusion protein following the standard protocol. Anti-EGFR and Anti-phospho-EGFR was purchased from Cell Signaling Technology (CST, USA). Anti-ANXA2 was from Novus Biotechnology (Novus Biotechnology, USA). Anti-phospho-ANXA2 was from Santa Cruz (Santa Cruz, CA, USA). A2PP (1st to 30th aa) and various truncated A2PP peptides, including A2PP-Nd4 (5th to 30th aa), A2PP-Nd8 (9th to 30th aa), A2PP-Nd12 (13rd to 30th aa), A2PP-Nd16 (17th to 30th aa), A2PP-Cd4 (1st to 26th aa), A2PP-Cd8 (1st to 22nd aa), A2PP-Cd12 (1st to 18th aa), A2PP-Cd16 (1st to 14th aa), together with random control peptides (ConP) were synthesized by Sbsbio (Beijing, China). Antimicrobials mycoplasma I (MYCO I) and mycoplasma II (MYCO II) were purchased from M&C GENE TECHNOLOGY (Beijing, China). Ciprofloxacin (CIP) was from Double-Crane Pharm (Beijing, China).

Detection of *M*. *hyorhinis* by Quantitative PCR (qPCR)

DNA was extracted from AGS or BGC823 cells after *M*. *hyorhinis* infection by DNA lysis buffer (50 mM Tris pH 8.5, 1 mM EDTA, 0.5% Tween-20, and 200 mg/L proteinase K) according to the standard protocol. qPCR was performed with 30 ng DNA and SYBR Green Real-time PCR 2×premix kit (Takara, Otsu, Japan) using Step One system from ABI (Foster City, CA, USA). The reaction programs and *p37*-specific primers (forward: 5′ -TATCTCATTGACCTTGACTAAC-3′ reverse: 5′ -ATTTTCGCCAATAGCATTTG-3′) were reported previously [22]. To compare *M*. *hyorhinis* DNA levels in cells, *GAPDH* (forward: 5′ -TGAAGGTCGGAGTCAACGG-3′ , reverse: 5′ -CCTGGAAGATGGTGATGGG-3′) was amplified as control and the data were analyzed using the $2^{-\Delta\Delta Ct}$ method. Primers were synthesized by Sangon (Shanghai, China).

Western Blotting

Cells were harvested from culture dish with 2×SDS loading buffer. Polyacrylamide gel electrophoresis (SDS-PAGE) and Western blotting were performed as previously described [23].

Cell ELISA

96-well were seeded with cells (1×10^4/well), followed by treatment of indicated peptides and infection of *M*. *hyorhinis* for 24 hr. Cells were immobilized with 0.05% glutaraldehyde for 10 min, then cell ELISA was performed as described previously [24].

Solid-Phase Binding Assay and Pull-Down Assay

Recombinant GST-p37 and GST proteins were generated and purified as previously described [17]. GST-p37 and GST were diluted in buffer (0.1M Na_2CO_3, 0.1M $NaHCO_3$, PH 9.6) and coated in 96-wells plates at 4°C overnight. The plates were washed by PBS for three times and blocked by 5% skimmed milk/PBS at room temperature (RT) for 2 hr. After washing with PBS, indicated concentrations of biotin-conjugated A2PP (synthesized by Sbsbio) was added and incubated at RT for 2 hr. After washing with PBST for 3 times, streptavidin-conjugated HRP (Baltimore Pike, West Grove, PA, USA) was added and incubated at RT for 30 min. After color development with Ortho-Phenylenediamine (Sigma), optical density at 490 nm (OD490) was recorded with a Microplate reader (Bio-rad 550). For pull-down assay, 100 ng GST-p37 or GST protein was co-incubated with 20 μM biotin-A2PP and streptavidin beads (GE Healthcare, Pittsburgh, PA, USA) in binding buffer (50 mM Tris-HCl pH 8.0, 150 mM NaCl, 0.5% NP-40, 0.5 mM DTT, 1 mM PMSF, and 1 × complete protease inhibitors) at 4°C overnight. The precipitates were washed with binding buffer for four times and analyzed by Western blotting.

Co-Immunoprecipitation

M. *hyorhinis* infected cells (BGC823 or AGS) were homogenized in lysis buffer (50 mM Tris-HCl pH 8.0, 150 mM NaCl, 1% Triton X-100, 0.5 mM DTT, 1 mM PMSF, and 1 × complete protease inhibitors) at 4°C for 10 min. After 12, 000 g centrifugation for 10 min at 4°C, supernatants were recovered. Protein lysates (500 μg) incubated with 20 μM A2PP or ConP, 1 μg anti-ANXA2 plus protein G sepharose beads (GE Healthcare) at 4°C overnight. Pre-immune IgG (1 μg) was used as control. Precipitated beads were washed with lysis buffer for four times, eluted in 2 × loading buffer, boiled, and analyzed by Western blotting.

Immunofluorescence Assay

Cells were seeded on coverslips and cultured overnight. The next day, cells were treated with indicated peptides and infected with *M. hyorhinis* for 24 hr. Then cells were washed with ice cold PBS for three times, fixed in 4% paraformaldehyde for 15 min at RT. After blocking for 1 hr in 5% BSA/PBS, cells were incubated with indicated antibodies at RT for 1 hr. Next, cells were washed for 3 times again with PBST and incubated with FITC or TRITC-labeled secondary antibodies for 30 min at RT. Following washing with PBS and counterstaining with DAPI, cells were mounted on 50% glycerol/PBS. A ZEISS LSM780 confocal microscope (ZEISS Microsystems, Oberkochen, Germany) was used to observe the localization of indicated proteins.

Cell Migration Assay

Transwell chamber with 8.0 μm pore membranes (Corning, NY, USA) was used in the cell migration assay. The bottom chamber was filled with 800 μL medium containing 10% FBS as chemoattractant. Cells were resuspended in serum-free medium containing *M. hyorhinis* plus A2PP or ConP, then were carefully transferred onto the top chamber of each Transwell apparatus at a density of 2×10^5 cells/mL (120 μL/chamber). Cells were allowed to migrate for 24 hr at 37°C. The top surface of each membrane was cleared of cells with a cotton swab. Cells penetrated to the bottom side of the membrane were fixed in cold methanol, stained with 0.1% crystal violet, and counted in nine randomly selected microscopic fields per well. Each sample was prepared in triplicate chambers and each experiment was repeated for at least 3 times.

Cell Proliferation Assay

Gastric cancer cells were seeded in 96-well culture plates at density of 5×10^3/100 μl/well in triplicates, and were treated with indicated reagents. Proliferation of cells were quantified by the cell confluence with a CloneSelect Imager (Molecular Devices, Sunnyvale, CA, USA).

Microarray Analysis and Real-Time RT-PCR

AGS and BGC823 cells (1×10^6 per 10 cm^2 plate) were treated with A2PP, CIP, MYCO I and MYCO II for 24 hr, washed with PBS, and harvested in Trizol reagent. RNA samples were examined in OE Biotechnology (Shanghai, China) by using Affymetrix GeneChip® PrimeView™ Human Gene Expression Array. Microarray data has been deposited in NCBI Gene Expression Omnibus (GEO) (accession no.GSE73777). Raw data was recorded by using Affymetrix GeneChip Command Console (version 4.0, Affymetrix). Next, Genespring software (version 12.5, Agilent Technologies) was employed to finish the basic analysis with the raw data. To begin with, the raw data was normalized with the RMA algorithm. Differentially expressed genes were then identified through fold change. The threshold set for up- and down-regulated genes was a fold change ≥ 2.0. Afterwards, GO and KEGG analysis were applied to select out genes that related to cell cycle and cell apoptosis. Real-time RT-PCR was used to validate the results of microarray and performed according to the manufacturers' instruction (SYBR, TOYOBO). Expression levels of related genes were normalized through *GAPDH* and the $2^{-\Delta\Delta CT}$ was used to calculate relative gene expression. The primers for real-time RT-PCR (*ATF*, forward, 5'-GAGTGGCGACAGGATAGAGC-3', reverse, 5'-TTTAGCCTCCCTCCCTTAGC-3'; *DDIT3*, forward, 5'-GCGCATGAAGGAGAAAGAAC-3', reverse, 5'-ACCATTCGGTCAATCAGAGC-3'; *CEBPB*, forward, 5'-AGCGACGAGTACAAGATCCG-3', reverse, 5'-AGCTGCTCCACCTTCTTCTG-3') were synthesized by Sangon.

Statistical Analysis

Data were presented as mean ± SD. The differences were analyzed by ANOVA using SPSS 11.0 software and $P < 0.05$ was considered statistically significant.

Microarray Accession Number

Microarray data has been deposited in NCBI Gene Expression Omnibus (GEO) (accession no. GSE73777).

Results

A2PP Has No Effect on the Viability or Migration of the Gastric Cancer Cells

In order to evaluate the role of ANXA2's N-terminal domain in *M. hyorhinis* infection, we firstly synthesized A2PP peptide corresponding to N-terminal of ANXA2 (Fig 1A). Biotin labeled A2PP was shown to specifically interact with GST-p37 in the solid phase binding assay (Fig 1B) and pull-down assay (Fig 1C). We noticed that A2PP didn't affect the morphology of AGS and BGC823 cells (Fig 1D). In addition, in concentrations less than 20 μM, A2PP didn't inhibit cell proliferation (Fig 1E), suggesting that A2PP has minimal toxicity to cells. EGFR has been implicated in regulating ANXA2 phosphorylation [17,25,26], while A2PP had no effects on the phosphorylations or protein levels of EGFR and ANXA2 [Fig 2A]. Moreover, A2PP had no effects on the migration of AGS and BGC823 cells (Fig 2B and 2C). Cellular localization of ANXA2 is regulated by its phosphorylation, which is critical for its function [17,27,28]. We found A2PP didn't change the subcellular localization of endogenous ANXA2 (Fig 2D). These results indicate that A2PP alone has no effects on malignant phenotypes or EGFR-ANXA2 signaling of gastric cancer cells.

A2PP Decreases *M. hyorhinis* Infection

Our previous work has shown that N-terminal of ANXA2 mediates *M. hyorhinis* infection [17]. As shown by the result of cell ELISA assay, we found that *M. hyorhinis* infection was blocked by A2PP in a dose-dependent manner in BGC823 and AGS cells, but ConP-treated cells were still highly infected by *M. hyorhinis* (Fig 3A). These results were supported by qPCR assays (Fig 3B). Consistently, A2PP also decreased protein levels of p37 protein in the Western blotting analysis (Fig 3C and 3D). Meanwhile, levels of phophos-AXNA2 and phophos-EGFR in the infected cells were down-regulated by treatment with A2PP (Fig 3C and 3E), while total levels of AXNA2 and EGFR were relatively stable (Fig 3C). In the immunofluorescence analysis, A2PP was found to inhibit *M. hyorhinis* infection-induced p37 accumulation and co-localization between p37 and ANXA2 (Fig 4A). In the co-immunoprecipitation assay with protein lysates from *M. Hyorhinis* infected cells, A2PP decreased the interaction between p37 and ANXA2 (Fig 4B). Together, these evidences supported the idea that A2PP could decrease *M. hyorhinis* infection and confirmed our previous discovery that the N-terminal of ANXA2 is required for mediating *M. hyorhinis* infection [17].

A2PP Suppresses *M. hyorhinis*-Induced Migration

Our previous studies suggested that *M. hyorhinis* infection could promote migration of gastric cancer cells [17, 23]. In this study, we noticed that *M. hyorhinis*-promoted migration of gastric cancer cells was not affected by ConP, but was dose-dependently inhibited by A2PP (Fig 5A and 5B). These results suggest that A2PP inhibited *M. hyorhinis*-induced migration, which was

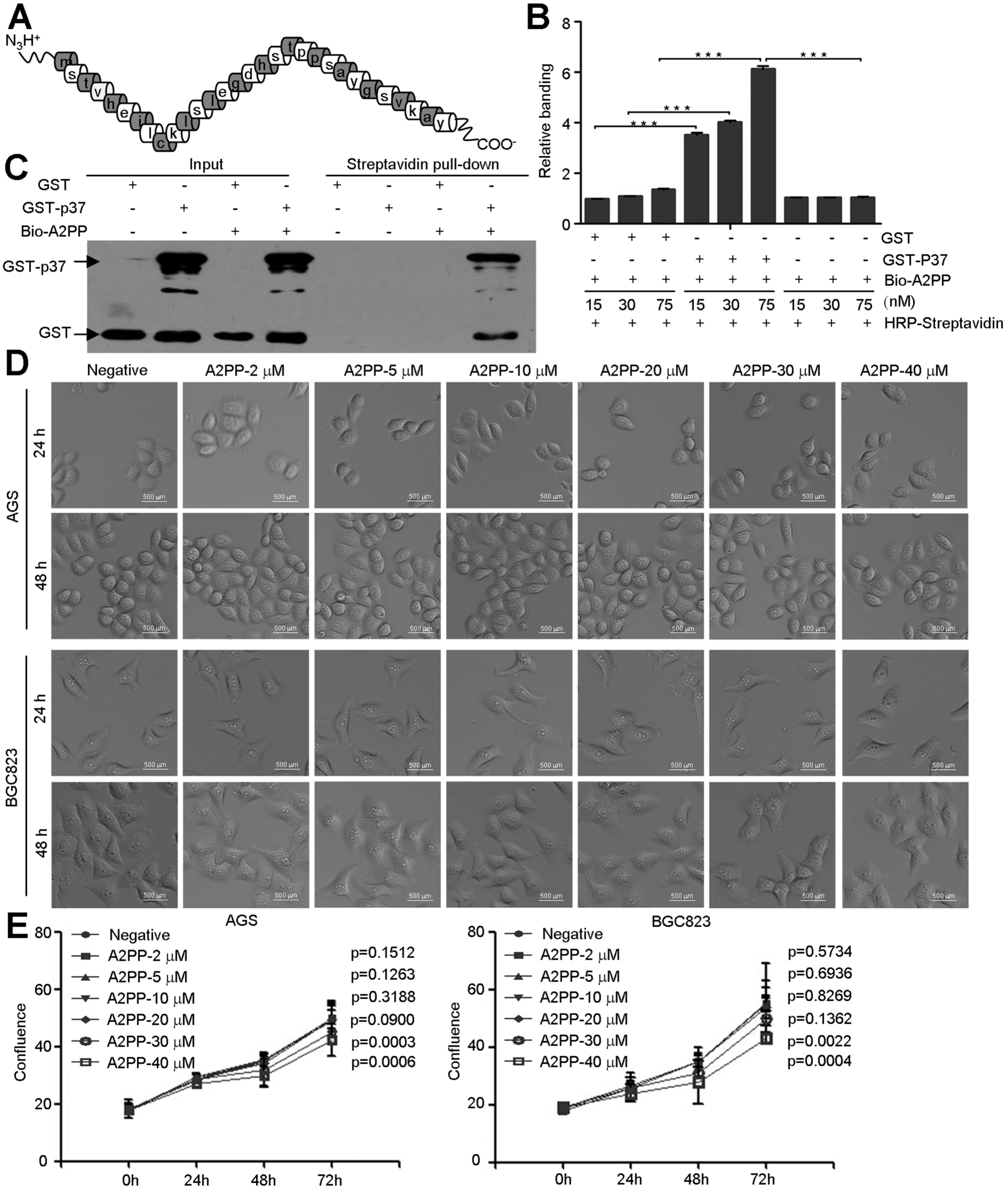

Fig 1. A2PP binds to p37 of *M. hyorhinis* and has minimal effect on the proliferation of gastric cancer cell. (A) Schematic diagram of amino acid sequence of N-terminal of ANXA2 polypeptide (A2PP). (B) Solid-phase binding assay. OD490 of 15 nM biotin-A2PP to GST was set as 1 and relative bindings were caculated. Mean ± SD of three independent assays with triplicate samples. ***, P < 0.001. (C) Streptavidin pull-down assays identified Biotin-A2PP as a GST-p37 binding polypeptide. (D) Cell morphology of AGS and BGC823 following indicated concentrations of A2PP treatment for 24 hr and 48 hr. (E) Proliferation of gastric cancer cell lines (AGS and BGC823) treated with increasing concentration of A2PP for 72 hr. Mean ± SD from three experiments with triplicate samples.

doi:10.1371/journal.pone.0147776.g001

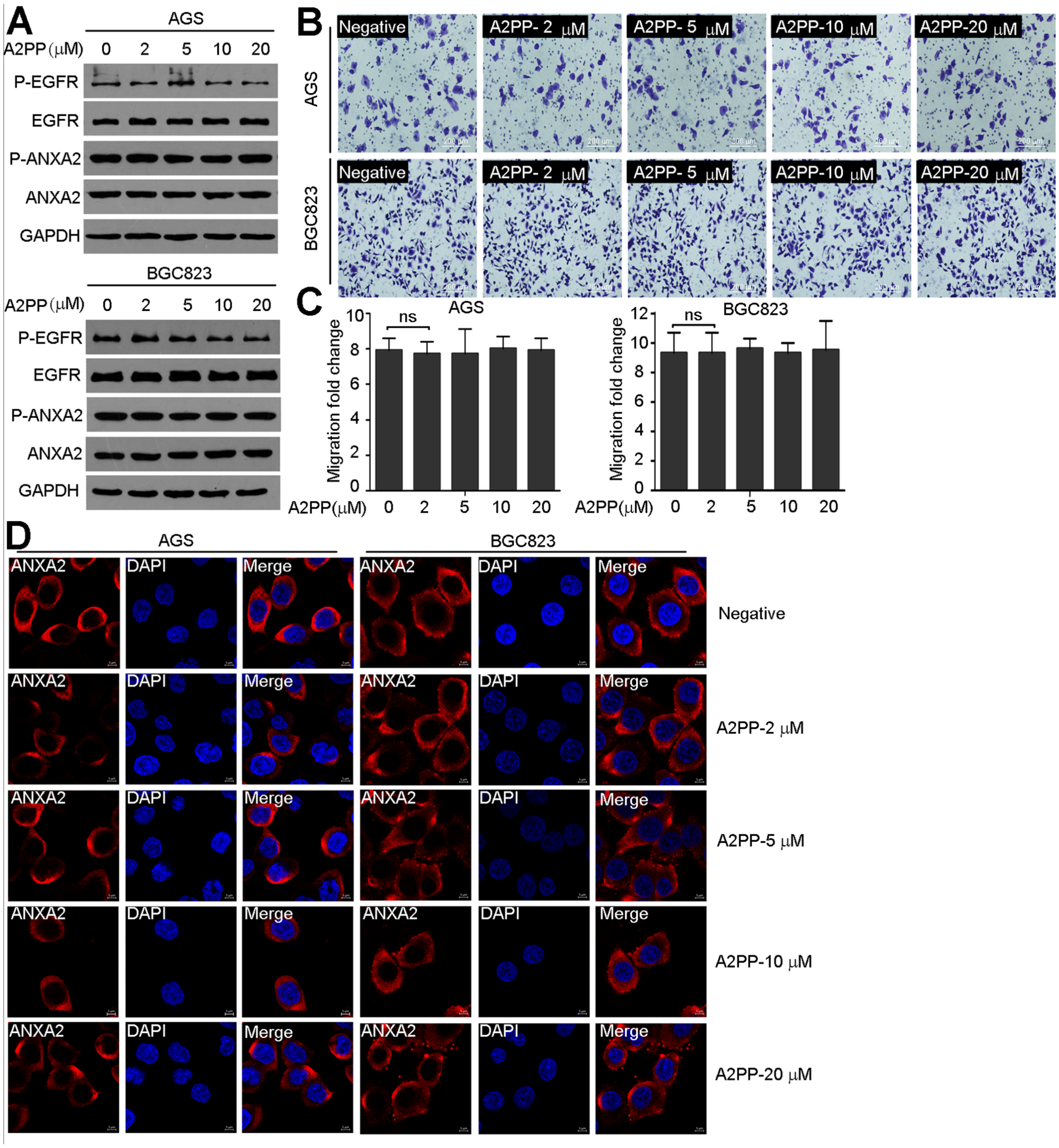

Fig 2. A2PP has minimal effects on EGFR-ANXA2 signaling, migration of gastric cancer cells, or the localization of ANXA2. (A) Western blotting of phospho-EGFR, phospho-ANXA2, EGFR, and ANXA2 from AGS and BGC823 cells treated with A2PP for 24 hr. GAPDH was used as loading control. (B) Representative images of migration of AGS and BGC823 cells treated with A2PP for 24 hr. Scale bars, 200 μm. (C) Statistical summary of migration assay. Mean ± SD from three experiments with triplicate samples. ns, no significance. (D) Immunofluorescence of ANXA2 localization (red) in AGS and BGC823 cells treated with A2PP for 24 hr. Scale bars, 5 μm.

doi:10.1371/journal.pone.0147776.g002

associated with its ability to decrease infection-promoted phosphorylations of EGFR and ANXA2 (Fig 3C and 3E).

A2PP Has Essential Motif to Decrease *M*. *hyorhinis* Infection

To characterize the critical motif of A2PP, we sought to determine whether diverse truncated peptides of A2PP (Fig 6A) could reduces *M*. *hyorhinis* infection. In the qPCR analysis, A2PP

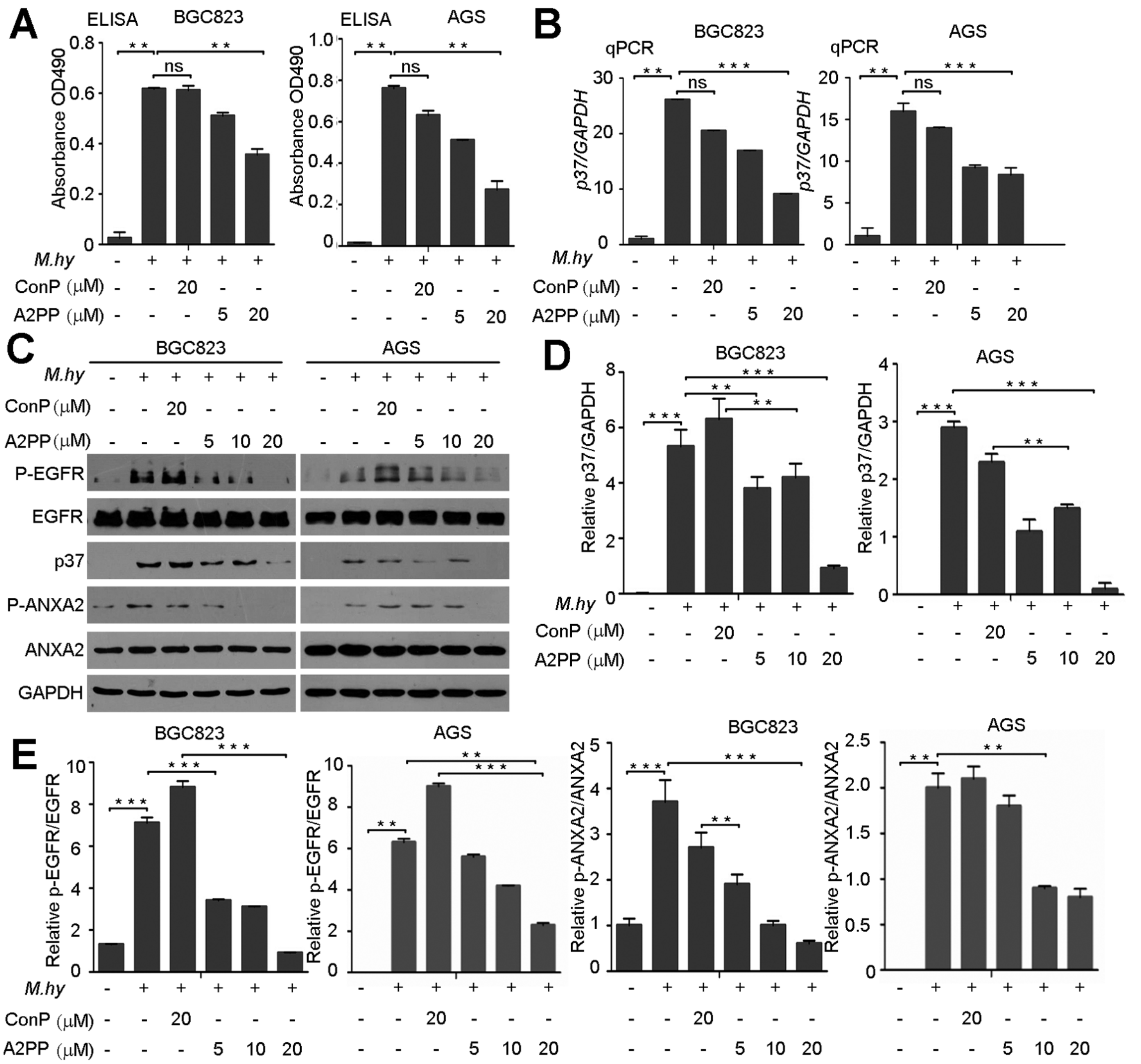

Fig 3. A2PP reduces *M. hyorhinis* infection. Cell ELISA analysis of p37 (OD 490 nm) of p37 protein in AGS and BGC823 cells infected with 10^5 CCU (color changing units)/ml of *M. hyorhinis* and treated with A2PP or ConP for 24 hr. *M. hy*, *M. hyorhinis*. Mean ± SD from 3 experiments with triplicate for each sample. (B) Quantitative PCR (qPCR) analysis of *p37* in AGS and BGC823 cells infected and treated as in (A). Mean ± SD from 3 experiments with triplicate for each sample. (C) Western blotting of p37, p-EGFR, EGFR, p-ANXA2 and ANXA2 from AGS and BGC823 cells treated as in (A). (D) Quantification of p37 protein levels of in (C). Levels of p37 were normalized to those of GAPDH. Mean ± SD from 3 independent experiments. (E) Quantification of p-EGFR and p-ANXA2 levels in (C). Levels of p-EGFR or p-ANXA2 were normalized to those of EGFR or ANXA2. Mean ± SD from 3 independent experiments. **, P < 0.01; **, P < 0.001; n.s, no significance.

doi:10.1371/journal.pone.0147776.g003

and truncated peptides A2PP-Nd4, Nd8, Nd12, Cd4, Cd8, and Cd12 reduced *M. hyorhinis* infection in gastric cancer cells, but A2PP-Nd16 and A2PP-Cd16 failed to decrease *M. hyorhinis* infection (Fig 6B). Similar pattern of inhibition was obtained from Western blotting assay (Fig 6C). These results suggest the existence of a specific motif of A2PP is essential for its ability to reduce *M. hyorhinis* infection. To further map the core sequences of this potential motif, we synthesized the two peptides corresponding to 11th -20th aa (termed as A2PP-10aa) and 13th -18th aa (termed as A2PP-6aa) (Fig 7A). In qPCR assay, both peptides could decrease *M. hyorhinis* infection (Fig 7B), which was supported by Western blotting assay (Fig 7C). Notably, the inhibitory effects of A2PP-10aa were better than those of A2PP-6aa, but the effects of both

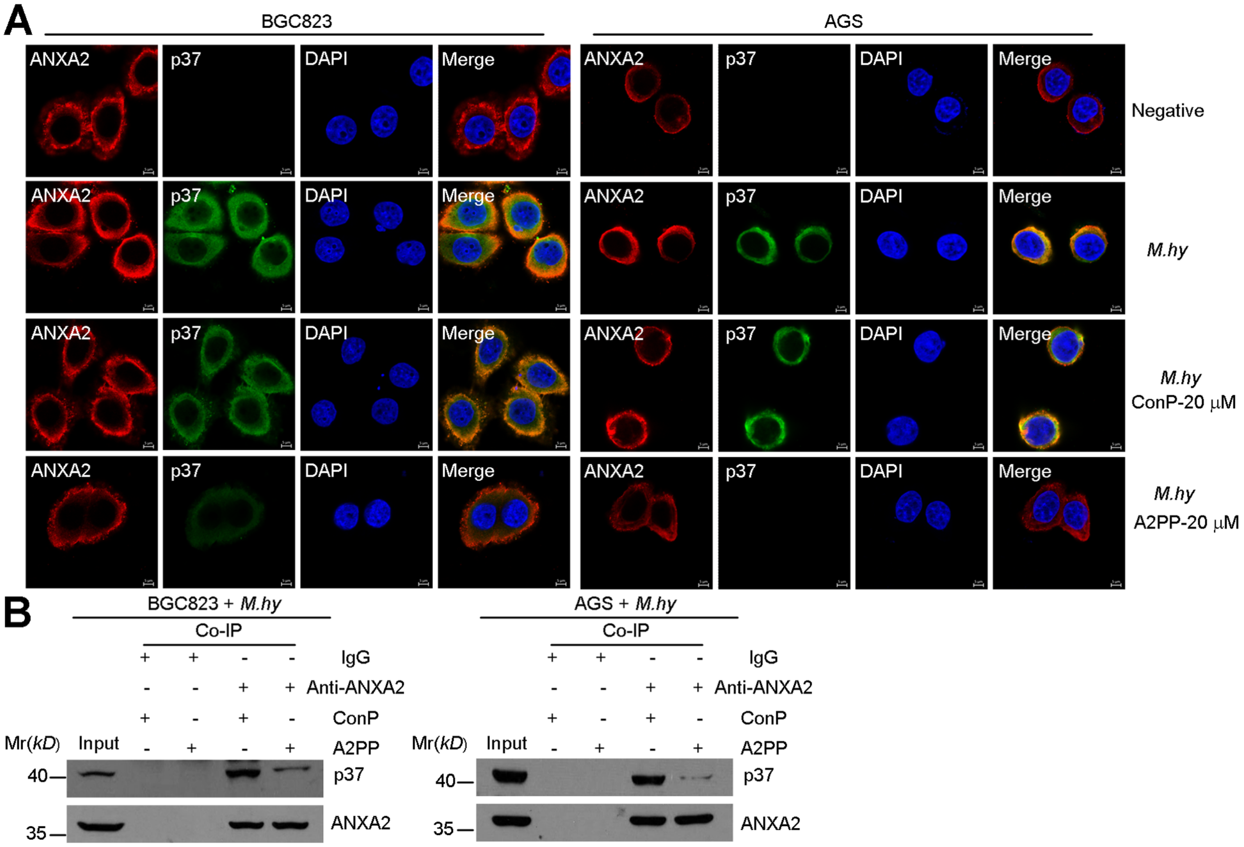

Fig 4. A2PP suppresses the interaction between ANXA2 and p37. (A) Localizations of ANXA2 (red) and p37 (green) in BGC823 and AGS cells infected with 10^5 CCU/ml of *M*. *hyorhinis* and treated with A2PP or ConP for 24 hr. Colocalization was shown by merged signals (yellow). Scale bars, 5 μm. (B) Co-immunoprecipitation assay to validate ANXA2-p37 interaction in BGC823 and AGS cells infected with *M*. *hyorhinis* and treated with A2PP or ConP for 24 hr.

doi:10.1371/journal.pone.0147776.g004

peptides were less robust as those of A2PP (Fig 7B and 7C). Thesefore, the central sequences (11th -20th) of A2PP could be the essential motif to inhibit *M*. *hyorhinis* infection.

Comparison of A2PP with Other Drugs in Preventing *M*. *hyorhinis* Infection

M. *hyorhinis* was one of the main pollution sources of cell culture [13,14,15]. The best way to eliminate *M*. *hyorhinis* infection is to discard cells and quickly sterilize all the contacted vessels, but some infected cells couldn't be replaced in several cases. Consequently, it is imperative to use specific antimicrobial approaches to prevent or eliminate *M*. *hyorhinis* infection. There have been different antimicrobial agents for preventing *M*. *hyorhinis* from infecting. For example, Ciprofloxacin (CIP) could eradicate *M*. *hyorhinis* in a suitable concentration [29]. Two antimicrobial components named as mycoplasma I (MYCO I) and mycoplasma II (MYCO II) could reduce *M*. *hyorhinis* infection by blocking its protein and nucleic acid synthesis. However, some concerns such as low sensitivity, strong resistance and high toxicity may limit their applications in cell culture. Based on these considerations, we believe it is necessary to compare the efficiencies of A2PP, CIP, MYCO I and MYCO II in preventing *M*. *hyorhinis* infection. By Western blotting and qPCR assays, we found that A2PP's inhibitory effect on *M*. *hyorhinis* infection was similar to that of MYCO I, but was better than those of CIP and MYCO II (Fig 8A and 8B). Interestingly, we found that A2PP could also efficiently eliminate the *M*. *hyorhinis*

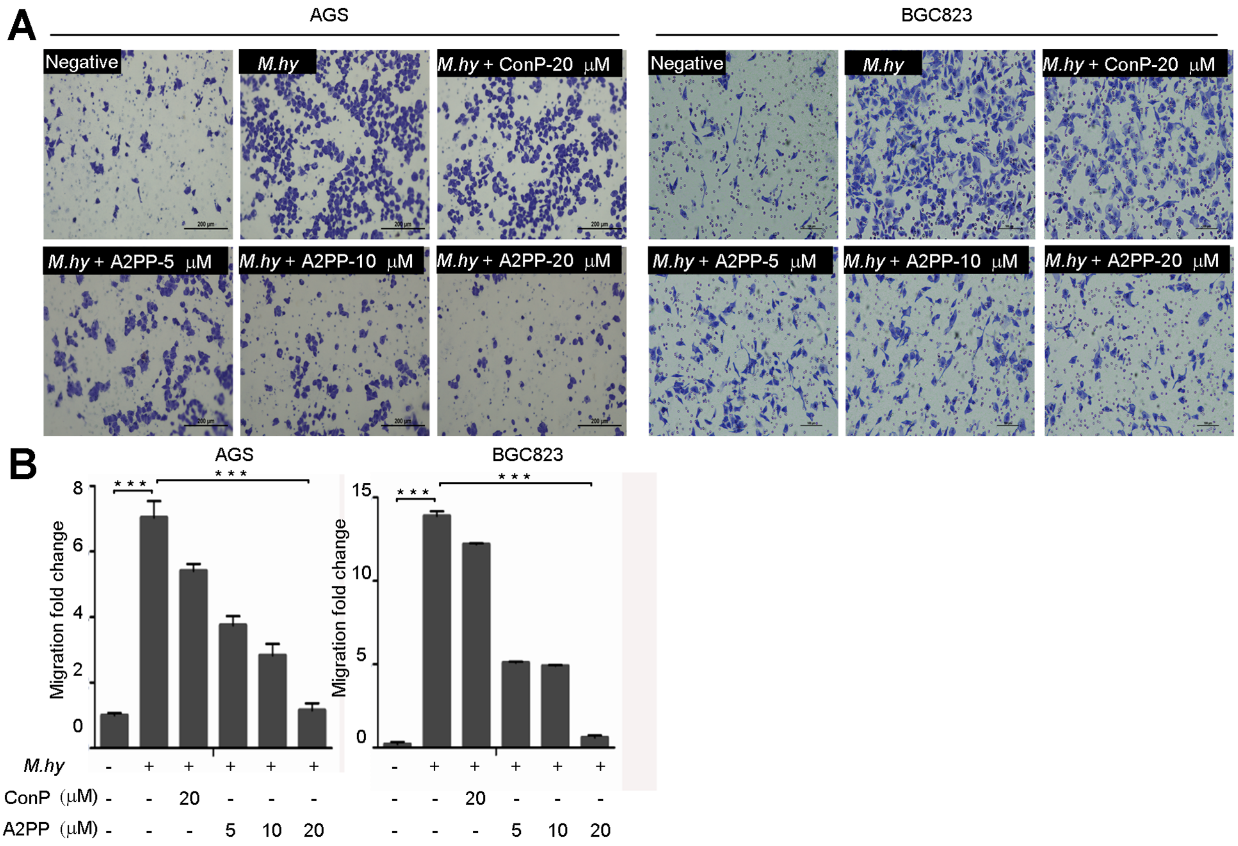

Fig 5. A2PP suppresses migration of gastric cancer cell induced by *M. hyorhinis* infection. (A) Migration of 10^5 CCU/ml of *M. hyorhinis*-infected AGS and BGC823 cells treated with indicated peptides for 24 hr. (B) Summary of migration assays (n = 3). Mean ± SD from 3 independent experiments with triplicate samples. ***, P<0.001.

doi:10.1371/journal.pone.0147776.g005

infection in heavily infected cells in the process of passages from P_1 to P_3 (Fig 8C and 8D). Additionally, compared with MYCO I and MYCO II, A2PP had less inhibitory effect on the cells proliferation (Fig 9A). To explore the mechanisms of A2PP, CIP, MYCO I and MYCO II's effects on cells proliferation, we performed microarray analysis and screened a subset of differentially expressed genes in A2PP, CIP, MYCO I and MYCO II treated cells. Quantitative RT-PCR analysis showed increased expression of apoptosis-related genes (*ATF5*, *DDIT3*, *CEBPB*) by MYCO I and MYCO II treatment, but little changes were observed in A2PP and CIP groups (Fig 9B). These results indicate that A2PP has less toxicity and better preventative potential in blocking *M. hyorhinis* infection in cultured cells.

Discussion

Mycoplasma infection and contamination are still prevalent today and bring considerable risks to patients and research quality. In recent years, several studies suggested that mycoplasma infection was also associated with tumorigenesis [3–8]. Mycoplasma infection could cause the DNA damage, affect gene expression, and disrupt the cell cycle checkpoint and apoptotic response [30]. *M. hyorhinis* was found in 56% of gastric cancers, 55.1% of colon cancers and 39.7% breast cancers tissues [20]. Besides, serological testing showed that 36% benign prostatic hyperplasia (BPH) and 52% prostatic cancer tissues were *M. hyorhinis* positive [4]. These studies suggest a possible association between *M. hyorhinis* infection and the occurrence of tumors

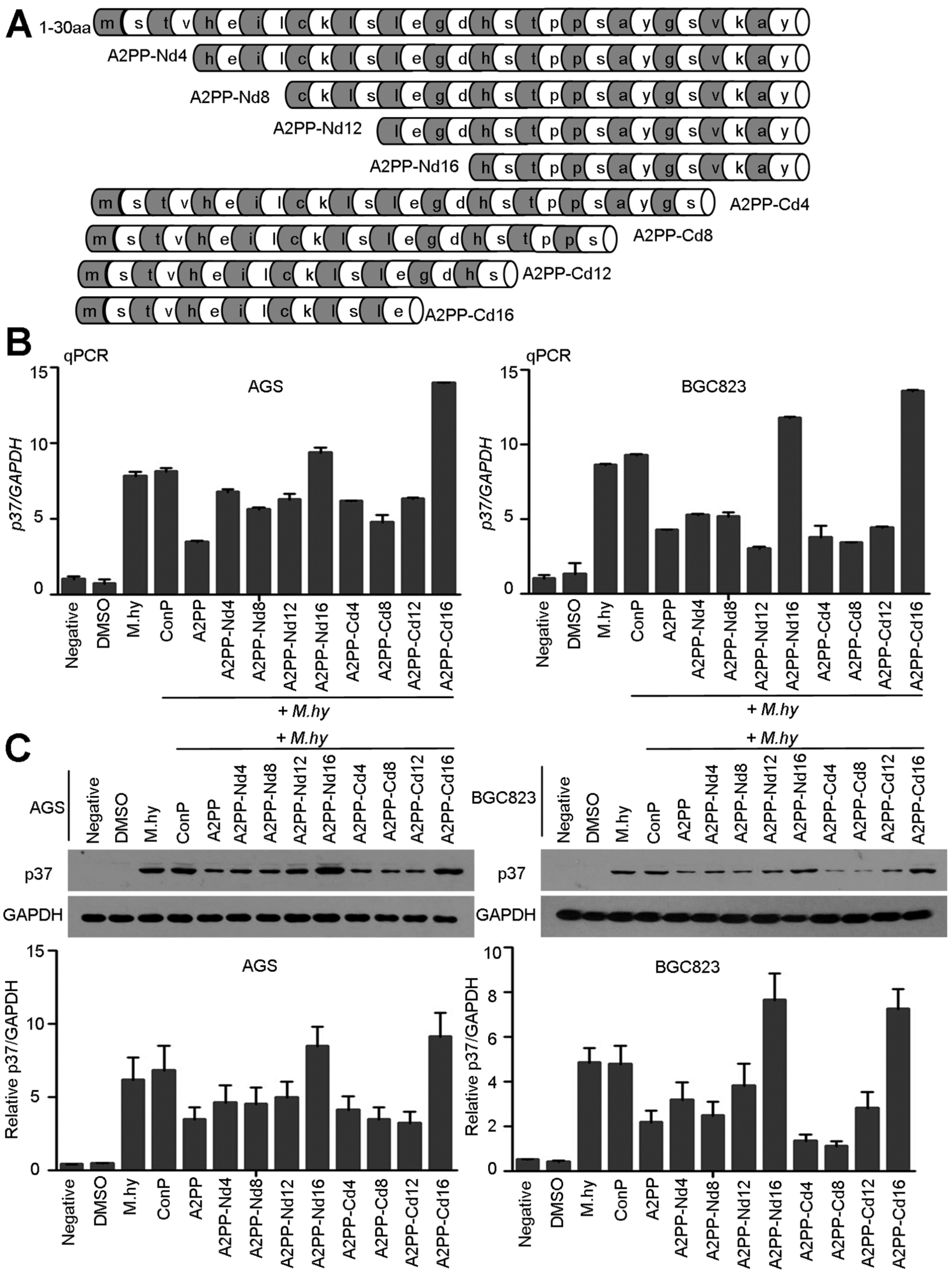

Fig 6. Effects of truncated A2PP peptides on *M. hyorhinis* infection. (A) Schematic diagram of truncated peptides of A2PP. (B) qPCR analysis of *p37* in AGS and BGC823 cells infected with 10^5 CCU/ml of *M. hyorhinis* and treated with 20 μM indicated peptides for 24 hr. Mean ± SD from 3 experiments with triplicate for each sample. (C) Western blotting of p37 from AGS and BGC823 cells infected and treated as in (B). Mean ± SD from 3 independent experiments.

doi:10.1371/journal.pone.0147776.g006

[4,20]. What's more, in the process of cell culture, the most common contamination sources are mycoplasma, bacteria and mold, but the contamination rate of mycoplasma is relatively higher [13]. Results from various groups have shown that the rate of mycoplasma contamination varied from 15 to 80%, some even reached 100% [15,31]. An analysis of DNA sequences

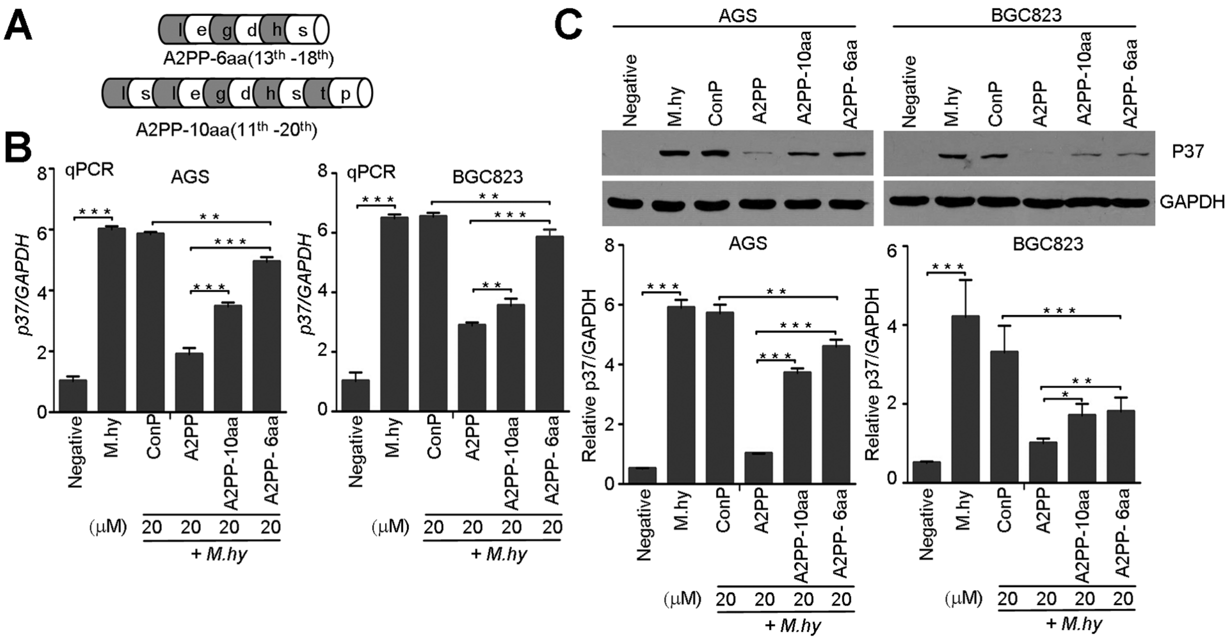

Fig 7. Specific sequence of A2PP decreases *M. hyorhinis* infection. Schematic diagram of two truncated forms of A2PP. (B) qPCR analysis of *p37* in AGS and BGC823 cells infected with 10^5 CCU/ml of *M. hyorhinis* and treated with 20 μM indicated peptides for 24 hr. Mean ± SD from 3 experiments with triplicate for each sample. (C) Western blotting of p37 from AGS and BGC823 cells infected and treated as in (B). Mean ± SD from 3 independent experiments. *, $P < 0.05$; **, $P < 0.01$; ***, $P < 0.001$.

doi:10.1371/journal.pone.0147776.g007

from 1000 genomes project implied that 7% of the samples were contaminated by mycoplasma [32]. However, preventing mycoplasma infection and contamination is difficult. Firstly, because of pleomorphic, plasticity, filterability and easy solubility, mycoplasmas could pass through microfiltration membrane easily, rendering them exist on the host cell surface or to be endocytosed by cells [1,13,33]. Secondly, mycoplasma lacks cell walls, rendering them insensitive to antibiotics which inhibit cell wall synthesis, such as penicillin. Effective antimicrobials do exist, but their continuous applications in cell culture is not recommended because of the toxicity to cells. Finally, mycoplasma is a atypical bacteria, causing difficulities in correct diagnosis [34].

Numerous anti-mycoplasma drugs have been developed, such as Erythromycin, Leucomycin, Roxithromycin, Ofloxacin, and Ciprofloxacin. However the adverse effects, toxicity, and resistance still bring trouble for pollution abatement [10,11,35–38]. Besides, continuous administration of these antibiotics couldn't completely eliminate mycoplasma for one or even more weeks [39,40]. Therefore, it is critical to find new ways that can eliminate the mycoplasma infection or contamination effectively and timely. The solution to this aim can probably be found through the characterization of molecular mechanisms underlying mycoplasma infection.

Our previous study found that the interaction of P37 protein with AXNA2 mediated *M. hyorhinis* infection [17]. In this study, we firstly synthesized the N-terminal polypeptide of ANXA2 (A2PP) and validated its specific interaction with GST-p37 protein in the solid-phase binding and streptavidin pull-down assays. Inspired by such interaction, we wondered whether A2PP could antagonize infection of *M. hyorhinis*. We found that A2PP, in an appropriate con-centration (20 μM), decreased *M. hyorhinis* infection, inhibited infection-promoted migration, and reduced infection-provoked the phosphorylations of EGFR and ANXA2 in gastric cancer cells. These effects were associated with decreased interaction between p37 and ANXA2. It

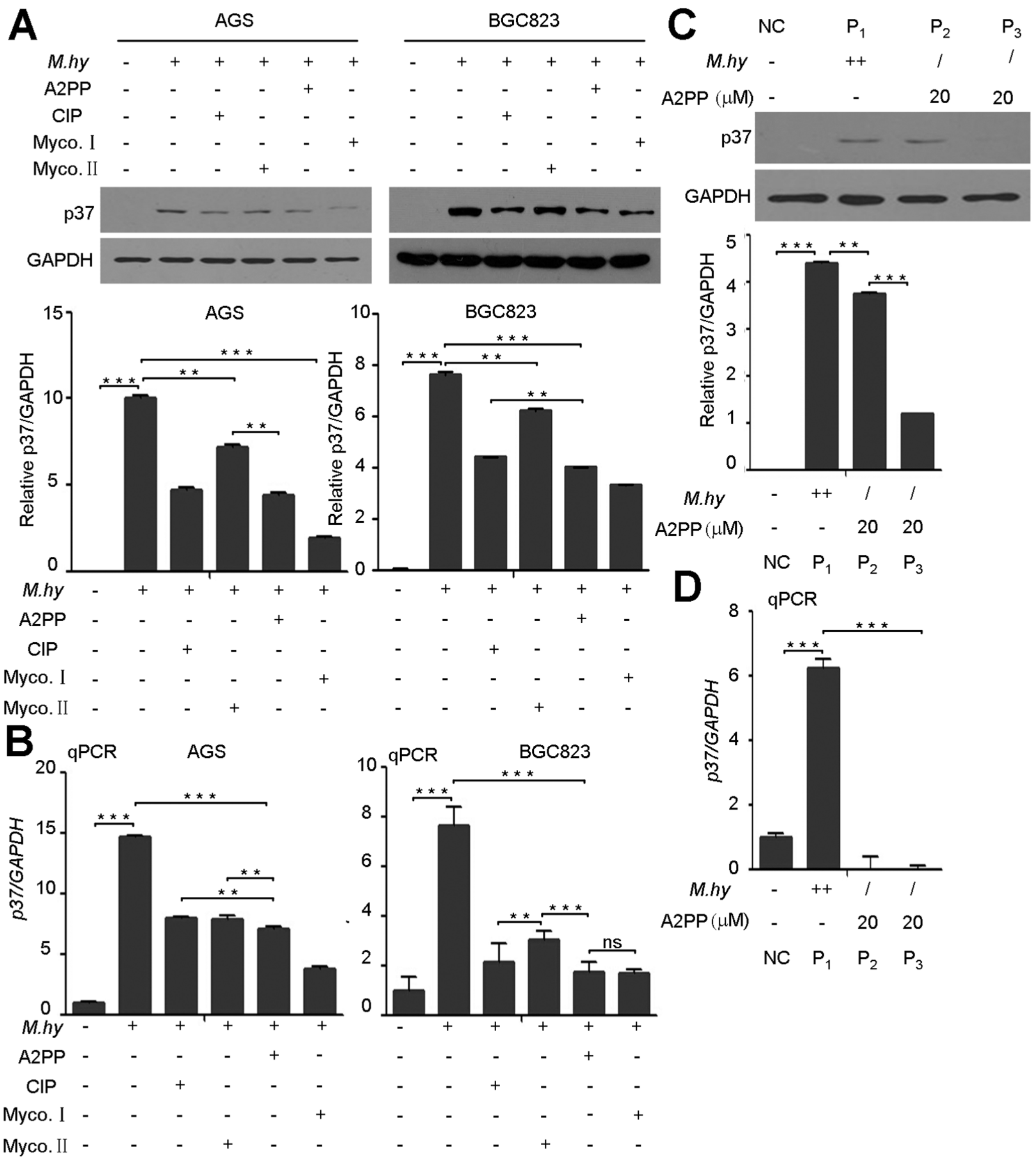

Fig 8. A2PP inhibits *M. hyorhinis* infection. (A) Western blotting of p37 from AGS and BGC823 cells infected with 10^5 CCU/ml of *M. hyorhinis* and treated with A2PP (20 μM), CIP (4 μg/ml), MYCO I (5 μg/ml), or MYCO II (10 μg/ml) for 24 hr. Mean ± SD from 3 independent experiments. (B) qPCR analysis of *p37* in AGS and BGC823 cells treated as in (A). Mean ± SD from 3 experiments with triplicate for each sample. (C) Western blotting of p37 from AGS cells infected with 10^5 CCU/ml of *M. hyorhinis* and treated with A2PP in the process of cell passages from P_1 to P_3. Mean ± SD from 3 independent experiments. (D) qPCR analysis of *p37* in AGS cells infected and treated as in (C). Mean ± SD from 3 experiments with triplicate samples. **, $P < 0.01$; ***, $P < 0.001$; ns, no significance.

doi:10.1371/journal.pone.0147776.g008

should be noted that A2PP alone exhibited minimal effects on proliferation of cells and phosphorylations of EGFR and ANXA2, suggesting that A2PP is likely to be a useful reagent to reduce *M. hyorhinis* infection. This notion was supported by comparisons of A2PP's and commercial antibiotics' effects on cell proliferation and *M. hyorhinis* infection, which showed that A2PP indeed had less toxicity to cells but strong ability to counteract infection.

Despite that A2PP, a 30 aa polypeptide, could decrease *M. hyorhinis* infection effectively, we still need to understand whether truncated forms of A2PP could achieve similar anti-*M. hyorhinis* ability. By using several truncated peptides of A2PP, we found that the central sequences

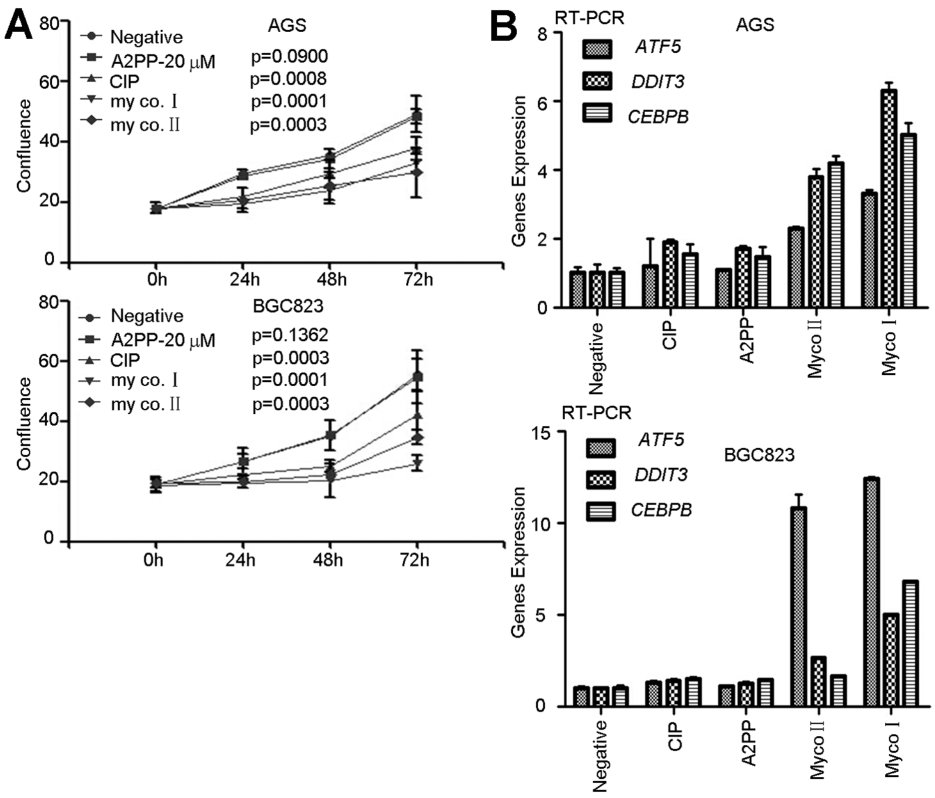

Fig 9. A2PP have a less cytotoxicity. (A) Proliferation of AGS and BGC823 treated with A2PP (20 μM), CIP (4 μg/ml), MYCO I (5 μg/ml), or MYCO II (10 μg/ml) for 72 hr. Mean ± SD from 4 independent experiments with triplicate samples. (B) RT-PCR analysis of *ATF5*, *DDIT3*, *CEBPB* in AGS and BGC823 cells treated with A2PP (10 μM), CIP (4 μg/ml), MYCO I (5 μg/ml), MYCO II (10 μg/ml) for 24 hr. Mean ± SD from 3 experiments with triplicate samples.

doi:10.1371/journal.pone.0147776.g009

(11th -20th) of A2PP could be the essential motif to inhibit *M. hyorhinis* infection. However, the inhibitory effects of truncated peptides were still not as strong as that of full length A2PP, suggesting that flanking sequences are essential to maintain proper structure to achieving efficient binding with p37. Further studies are required to find the effects of modification or/and mutation of this motif on inhibiting *M. hyorhinis* infection.

Acknowledgments

We thank and gratefully acknowledge Lin Meng, Dr. Zhihua Tian (all from Peking University Cancer Hospital & Institute) for providing critical reagents or technical assistance.

Author Contributions

Conceived and designed the experiments: SY CS. Performed the experiments: SY. Analyzed the data: SY LQ CS. Wrote the paper: SY LQ.

References

1. Maniloff J, Morowitz HJ. Cell Biology of the Mycoplasmas. Bacteriol Rev. 1972; 36:263–90. PMID: 4345848
2. Razin S, Yogev D, Naot Y. Molecular biology and pathogenicity of mycoplasmas. Microbiol Rev. 1998; 63:1094–156.

3. Namiki K, Goodson S, Porvasnik S, Allan RW, Iczkowski KA, Urbanek C, et al. Persistent Exposure to Mycoplasma Induces Malignant Transformation of Human Prostate Cells. PLoS One. 2009; 4:e6872. doi: 10.1371/journal.pone.0006872 PMID: 19721714
4. Urbanek C, Goodison S, Chang M, Porvasnik S, Sakamoto N, Li CZ, et al. Detection of antibodies directed at *M*. *hyorhinis* p37 in the serum of men with newly diagnosed prostate cancer. BMC Cancer. 2011; 11:233. doi: 10.1186/1471-2407-11-233 PMID: 21663671
5. Yang H, Qu L, Ma H, Chen L, Liu W, Liu C, et al. *Mycoplasma hyorhinis* infection in gastric carcinoma and its effects on the malignant phenotypes of gastric cancer cells. BMC Gastroenterol. 2010; 10:132. doi: 10.1186/1471-230X-10-132 PMID: 21062494
6. Paton GR, Jacobs JP, Perkins FT. Chromosome changes in human Diploid-cell cultures infected with mycoplasmas. Nature. 1965; 207:43–5. PMID: 5866523
7. Tsai S, Wear DJ, Shih JW, Lo SC. Mycoplasmas and oncogenesis: persistent infection and multistage malignant transformation. Proc Natl Acad Sci USA. 1995; 92:10197–201. PMID: 7479753
8. Lo SC, Tsai S. Mycoplasmas and Human prostate cancer: An exciting but cautionary note. Oncotarget. 2011; 2:352–5. PMID: 21789784
9. Rogers MB. Mycoplasma and cancer: in search of the link. Oncotarget. 2011; 2:271–3. PMID: 21508438
10. Cazanave C, Manhart LE, Bebear C. *Mycoplasma genitalium*, an emerging sexually transmitted pathogen. Med Mal Infect. 2012; 42:381–92. doi: 10.1016/j.medmal.2012.05.006 PMID: 22975074
11. Chrisment D, Charron A, Cazanave C, Pereyre S, Bébéar C. Detection of macrolide resistance in *Mycoplasma genitalium* in France. J Antimicrob Chemother. 2012; 67:2598–601. doi: 10.1093/jac/dks263 PMID: 22773740
12. Roediger WE. Intestinal mycoplasma in Crohn's disease. Novartis Found Symp. 2004; 263:85–93. PMID: 15669636
13. Callaway E. Contamination hits cell work: Mycoplasma infestations are widespread and costing laboratories millions of dollars in lost research. Nature. 2014; 511:518. doi: 10.1038/511518a PMID: 25079533
14. Darin N, Kadhom N, Brière JJ, Chretien D, Bébéar CM, Rötig A, et al. Mitochondrial activities in human cultured skin fibroblasts contaminated by *Mycoplasma hyorhinis*. BMC Biochem. 2003; 4:15. PMID: 14596686
15. Drexler HG, Uphoff CC. Mycoplasma contamination of cell cultures: Incidence, sources, effects, detection, elimination, prevention. Cytotechnology. 2002; 39:75–90. doi: 10.1023/A:1022913015916 PMID: 19003295
16. Olarerin-George AO, Hogenesch JB. Assessing the prevalence of mycoplasma contamination in cell culture via a survey of NCBI's RNA-seq archive. Nucleic Acids Res. 2015; 43:2535–42. doi: 10.1093/nar/gkv136 PMID: 25712092
17. Duan H, Chen L, Qu L, Yang H, Song SW, Han Y, et al. *Mycoplasma hyorhinis* infection promotes NF-kB-dependent migration of gastric cancer cells. Cancer Res. 2014; 74:5782–94. doi: 10.1158/0008-5472.CAN-14-0650 PMID: 25136068
18. Hayflick L, Stinebring WR. Intracellular growth of pleura pneumonia like organisms (PPLO) in tissue culture and in ovo. Ann NY Acad Sci. 1960; 79:433–49. PMID: 14400338
19. Taylor G, Taylor-Robinson D, Slavin G. Effect of immunosuppression on arthritis in mice induced by *Mycoplasma pulmonis*. Ann Rheum Dis. 1974; 33:376–84. PMID: 4547195
20. Huang S, Li JY, Wu J, Meng L, Shou CC. Mycoplasma infections and different human carcinomas. World J Gastroenterol. 2001; 7:266–9. PMID: 11819772
21. Ning JY, Sun GX, Huang S, Ma H, An P, Meng L, et al. Identification of antigens by monoclonal antibody PD4 and its expression in Escherichia coli. World J Gastroenterol. 2003; 9:2164–8. PMID: 14562370
22. Tocqueville V, Ferré S, Phuc Nguyen NH, Kempf I, Marois-Créhan C. Multilocus sequence typing of *Mycoplasma hyorhinis* strains identified by a real-time TaqMan PCR assay. J Clin Microbiol. 2014; 52:1664–71. doi: 10.1128/JCM.03437-13 PMID: 24622092
23. Duan H, Qu L, Shou C. Activation of EGFR-PI3K-AKT signaling is required for *Mycoplasma hyorhinis*-promoted gastric cancer cell migration. Cancer Cell Int. 2014; 14:135. doi: 10.1186/s12935-014-0135-3 PMID: 25505372
24. Takei A, Huang Y, Lopes-Virella MF. Expression of adhesion molecules by human endothelial cells exposed to oxidized low density lipoprotein. Influences of degree of oxidation and location of oxidized LDL. Atherosclerosis. 2001; 154:79–86. PMID: 11137085

25. Shetty PK, Thamake SI, Biswas S, Johansson SL, Vishwanatha JK. Reciprocal regulation of annexin A2 and EGFR with Her-2 in Her-2 negative and herceptin-resistant breast cancer. PLoS One. 2012; 7: e44299. doi: 10.1371/journal.pone.0044299 PMID: 22957061

26. Dziduszko A, Ozbun MA. Annexin A2 and S100A10 regulate human papillomavirus type 16 entry and intracellular trafficking in human keratinocytes. J Virol. 2013; 87:7502–15. doi: 10.1128/JVI.00519-13 PMID: 23637395

27. Morel E, Gruenberg J. Annexin A2 binding to endosomes and functions in endosomal transport are regulated by tyrosine 23 phosphorylation. J Biol Chem. 2009; 284:1604–11. doi: 10.1074/jbc.M806499200 PMID: 18990701

28. Wang CY, Lin CF. Annexin A2: its molecular regulation and cellular expression in cancer development. Dis Markers. 2014; 2014:308976. doi: 10.1155/2014/308976 PMID: 24591759

29. Schmitt K, Däubener W, Bitter-Suermann D, Hadding U. A safe and efficient method for elimination of cell culture mycoplasmas using ciprofloxacin. J Immunol Methods. 1988; 109:17–25. PMID: 3282011

30. Sun G, Xu X, Wang Y, Shen X, Chen Z, Yang J, et al. *Mycoplasma pneumoniae* Infection Induces Reactive Oxygen Species and DNA Damage in A549 Human Lung Carcinoma Cells. Infect Immun. 2008; 76:4405–13. doi: 10.1128/IAI.00575-08 PMID: 18663006

31. Rottem S, Kornspan JD, Kosower NS. Contamination of tissue cultures by mycoplasmas. In: Ceccherini-Nelli L, Matteoli B,editors. Biomedical Tissue Culture. Rijeka: In Tech; 2012. pp. 35–58.

32. Langdon WB. Mycoplasma contamination in the 1000 Genomes Project. BioData Min. 2014; 7:3. doi: 10.1186/1756-0381-7-3 PMID: 24872843

33. Maniloff J. Electron microscopy of small cells: *Mycoplasma hominis*. J Bacteriol. 1969; 100:1402–8. PMID: 5361221

34. Whitson WJ, Ball PA, Lollis SS, Balkman JD, Bauer DF. Postoperative *Mycoplasma hominis* infections after neurosurgical intervention. J Neurosurg Pediatr. 2014; 14:212–8. doi: 10.3171/2014.4.PEDS13547 PMID: 24856879

35. Taylor-Robinson D, Jensen JS. *Mycoplasma genitalium*: from chrysalis to multicolored butterfly. Clin Microbiol Rev. 2011; 24:498–514. doi: 10.1128/CMR.00006-11 PMID: 21734246

36. Twin J, Jensen JS, Bradshaw CS, Garland SM, Fairley CK, Min LY, et al. Transmission and selection of macrolide resistant *Mycoplasma genitalium* infections detected by rapid high resolution melt analysis. PLoS One. 2012; 7:e35593. doi: 10.1371/journal.pone.0035593 PMID: 22532861

37. Tagg KA, Jeoffreys NJ, Couldwell DL, Donald JA, Gilbert GL. Fluoroquinolone and macrolide resistance-associated mutations in *Mycoplasma genitalium*. J Clin Microbiol. 2013; 51:2245–9. doi: 10.1128/JCM.00495-13 PMID: 23658265

38. Krausse R, Schubert S. In-vitro activities of tetracyclines, macrolides, fluoroquinolones and clindamycin against *Mycoplasma hominis* and Ureaplasma ssp. isolated in Germany over 20 years. Clin Microbiol Infec. 2010; 16:1649–55.

39. Hendershot L, Levitt D. Effects of mycoplasma contamination on immunoglobulin biosynthesis by human B lymphoblastoid cell lines. Infect Immun. 1985; 49:36–9. PMID: 3924831

40. Gurney T Jr, Woolf MJ, Abplanalp LJ, McKittrick NH, Dietz JN, Cole BC, et al. Elimination of *Mycoplasma hyorhinis* infections from four cell lines. In Vitro. 1981; 17:993–6. PMID: 7319531
